# Neutrophil-inspired photothermo-responsive drug delivery system for targeted treatment of bacterial infection and endotoxins neutralization

**DOI:** 10.1186/s40824-023-00372-z

**Published:** 2023-04-15

**Authors:** Chengnan Li, Yingying Gan, Zongshao Li, Mengjing Fu, Yuzhen Li, Xinran Peng, Yongqiang Yang, Guo-bao Tian, Yi Yan Yang, Peiyan Yuan, Xin Ding

**Affiliations:** 1 School of Pharmaceutical Science (Shenzhen), Shenzhen Campus of Sun Yat-sen University, Shenzhen, 518107 PR China; 2 Center for Pathogen Research, West China Hospital, Sichuan University, Chengdu, 610041 China; 3 Department of Immunology, School of Medicine, Sun Yat-sen University, Shenzhen, 518107 China; 4 Bioprocessing Technology Institute (BTI), Agency for Science, Technology and Research (A*STAR), 20 Biopolis Way, Centros #06-01, Singapore, 138668 Republic of Singapore

**Keywords:** Neutrophil membrane, Photothermal, Endotoxin neutralization, *Pseudomonas aeruginosa*, Drug delivery

## Abstract

**Background:**

*P. aeruginosa*, a highly virulent Gram-negative bacterium, can cause severe nosocomial infections, and it has developed resistance against most antibiotics. New therapeutic strategies are urgently needed to treat such bacterial infection and reduce its toxicity caused by endotoxin (lipopolysaccharide, LPS). Neutrophils have been proven to be able to target inflammation site and neutrophil membrane receptors such as Toll-like receptor-4 (TLR4) and CD14, and exhibit specific affinity to LPS. However, antibacterial delivery system based on the unique properties of neutrophils has not been reported.

**Methods:**

A neutrophil-inspired antibacterial delivery system for targeted photothermal treatment, stimuli-responsive antibiotic release and endotoxin neutralization is reported in this study. Specifically, the photothermal reagent indocyanine green (ICG) and antibiotic rifampicin (RIF) are co-loaded into poly(lactic-co-glycolic acid) (PLGA) nanoparticles (NP-ICG/RIF), followed by coating with neutrophil membrane to obtain antibacterial delivery system (NM-NP-ICG/RIF). The inflammation targeting properties, synergistic antibacterial activity of photothermal therapy and antibiotic treatment, and endotoxin neutralization have been studied in vitro. A *P. aeruginosa*-induced murine skin abscess infection model has been used to evaluate the therapeutic efficacy of the NM-NP-ICG/RIF.

**Results:**

Once irradiated by near-infrared lasers, the heat generated by NP-ICG/RIF triggers the release of RIF and ICG, resulting in a synergistic chemo-photothermal antibacterial effect against *P. aeruginosa* (~ 99.99% killing efficiency in 5 min). After coating with neutrophil-like cell membrane vesicles (NMVs), the nanoparticles (NM-NP-ICG/RIF) specifically bind to inflammatory vascular endothelial cells in infectious site, endowing the nanoparticles with an infection microenvironment targeting function to enhance retention time. Importantly, it is discovered for the first time that NMVs-coated nanoparticles are able to neutralize endotoxins. The *P. aeruginosa* murine skin abscess infection model further demonstrates the in vivo therapeutic efficacy of NM-NP-ICG/RIF.

**Conclusion:**

The neutrophil-inspired antibacterial delivery system (NM-NP-ICG/RIF) is capable of targeting infection microenvironment, neutralizing endotoxin, and eradicating bacteria through a synergistic effect of photothermal therapy and antibiotic treatment. This drug delivery system made from FDA-approved compounds provides a promising approach to fighting against hard-to-treat bacterial infections.

**Supplementary Information:**

The online version contains supplementary material available at 10.1186/s40824-023-00372-z.

## Introduction

*Pseudomonas aeruginosa* (*P. aeruginosa*) is one of the most dangerous bacteria that has been included in the list of 12 superbugs by the World Healthcare Organization (WHO). It is responsible for 10–20% nosocomial infections worldwide, [1, 2] causing serious infections such as pneumonia, skin and soft tissue infections, and urine tract infection etc. [1–6]. The multidrug resistance of *P. aeruginosa* resulted from various resistance mechanisms, such as efflux pumps, low outer membrane permeability and formation of biofilm, often leads to treatment failure of conventional antibiotics [5, 7–10]. Moreover, the lipopolysaccharide (LPS), also known as endotoxin, located on the Gram-negative bacterial outer membrane is a major virulence of *P. aeruginosa* infection. Once released in the process of cell proliferation, cell death or antibiotic treatment, LPS can cause inflammatory cytokine storms and subsequently result in sepsis [11–13]. Therefore, novel antimicrobial strategies that can kill the *P. aeruginosa* and neutralize endotoxin are of great need.

Various antimicrobial strategies such as photothermal therapy (PTT), photodynamic therapy (PDT) and the combination of PTT or PDT with antibiotic treatment, have been studied to treat *P. aeruginosa* infections and showed promise [14–16]. However, the lack of targeting and endotoxin neutralization functions could lead to toxicity and failure of the aforementioned approaches, preventing them from further clinical applications. Cell membrane-coated nanoparticles have recently emerged as a functional therapeutic platform for various applications [17–22]. Neutrophils, as the most abundant type of leucocytes in the body, participate in various inflammatory activities and provide the first line of immune defenses against invading pathogens or tissue damages, [23–25] and neutrophil-derived membrane has been used for inflammation-targeted delivery [23, 26–30]. In an inflammatory response, neutrophils are recruited to the inflammatory site *via* a cascade process, in which the adhesive proteins presented on neutrophils, such as β2 integrin, play a pivotal role for the adhesion with inflamed vascular endothelial cells [31, 32]. Meanwhile, as an important cell that participates in LPS-induced inflammation, neutrophil membrane receptors, such as Toll-like receptor-4 (TLR4) and CD14, exhibit specific affinity to LPS for the activation of the NF-κB-mediated inflammatory pathway [33, 34]. Inspired by the inflammatory cascade, we presume that neutrophil cell membrane-coated nanoparticles could target bacteria-infected site that is usually accompanied with inflammatory response, and may also neutralize the endotoxin (LPS) due to the specific binding of membrane receptors to LPS.

**Scheme 1:**
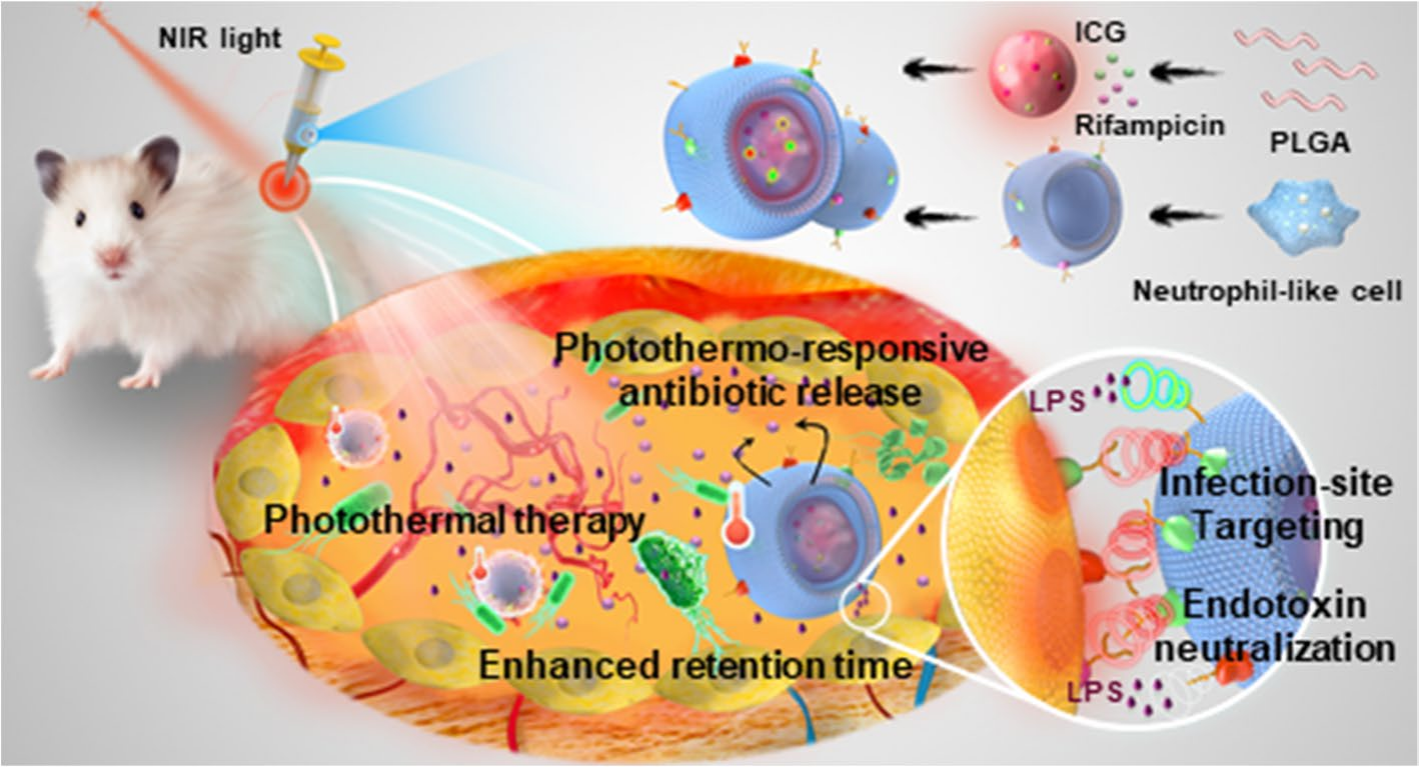
Schematic illustration of neutrophil-like cell membrane-coated photothermal drug delivery system for the combination treatment of *P. aeruginosa* infection

Herein, we report the neutrophil-like cell membrane-coated photothermal drug delivery system for the combination treatment of *P. aeruginosa* infection (Scheme 1). In this system, biodegradable poly (lactic-co-glycolic acid) (PLGA) nanoparticles (NP) were used as drug carriers for the co-loading of photothermal reagent indocyanine green (ICG) and antibiotic rifampicin (RIF). Although rifampicin is typically used to treat Gram-positive bacteria, this drug can be repurposed to treat Gram-negative bacterial infections when it is combined with proper adjuvants or delivery systems that are able to enhance bacterial membrane permeability to rifampicin [35–37]. Under irradiation of near infrared (NIR) light, the elevated temperature induced by photothermal agent ICG can not only trigger the glass transition of PLGA and in turn release the loaded RIF, [38] but may also damage bacterial membrane and enhance the membrane permeability to RIF, leading to a synergistic chemo-photothermal therapy. Coating of neutrophil-like cell membrane provides the targeting delivery of encapsulated drugs to the inflammatory tissue with infection, thus confining most of the heating to the infectious site instead of normal tissues. In the meantime, the neutrophil-like cell membrane is expected to locally neutralize LPS released from bacterial cells, preventing LPS from causing further severe inflammatory responses. The neutrophil-like cell membrane-coated nanoparticles-loaded with ICG and RIF were prepared and characterized, followed by the in vitro evaluation of inflammation targeting using LPS-stimulated vascular endothelial cells and LPS neutralization effects. In vivo antibacterial and anti-virulence therapeutic efficacy of the targeted delivery systems were further examined *via* a *P. aeruginosa* murine skin abscess infection model.

## Materials and methods

### Materials

PLGA (50:50) (16 kDa) was obtained from Jinan Daigang Co., Ltd, Jinan, China. Indocyanine green was purchased from J&K scientific Co., Ltd, Beijing, China. Rifampicin was bought from Beijing Solarbio Science & Technology Co., Ltd, Beijing, China. DiO fluorescent dye and BacLight Live/dead kit were obtained from MedChemExpress, U.S.A. LPS was obtained from Sigma Aldrich, U.S.A. Polyvinyl alcohol (PVA) 0588, d-mannitol, Rhodamine B, sucrose, EDTA and SDS were bought from Aladdin, Shanghai, China. Tris-HCl buffer (pH 7.5), MTT cell viability kit and BCA protein assay kit were bought from Beyotime Biotechnology, Shanghai, China. LB medium and agar were obtained from BD Difco, U.S.A. RPMI 1640 cell culture medium supplemented with 10% fetal bovine serum and 1% Penicillin-Streptomycin and IMEM medium supplemented with 20% fetal bovine serum and 1% penicillin & streptomycin were purchased from Gibco, Thermo Fisher Scientific, U.S.A. IL-6 and TNF-α ELISA kits were obtained from NeoBioscience Co., Ltd. Shenzhen, China. Mouse anti-ICAM-1 antibody (ab171123) and rabbit anti-integrin β1 monoclonal antibody (ab179471) were bought from Abcam, U.K. Rabbit anti-integrin β2 polyclonal antibody (#47598) was bought from cell signing technology (CST), U.S.A. Rabbit anti-TLR4 polyclonal antibody (bs-20594R) and Rabbit anti-CD14 monoclonal antibody (bsm-52556R) were purchased from Bioss Biotechnology Co., Ltd, Beijing, China.

Bacterial strain, *P. aeruginosa* (ATCC No. 27853) and HL-60 (ATCC No. CCL-240), HUVEC (ATCC No. PCS-100-041), HEK293T (ATCC No. CRL-11268), HACAT (ATCC No. PCS-200-011) and RAW264.7 (ATCC No. TIB-71) cells were purchased from American Type Culture Collection (ATCC, U.S.A.).

### Instruments

The morphology of the nanoparticles was examined on a transmission electron microscopy (TEM, FEI Tecnai G2 Spirit, FEI, U.S.A.). Photothermal heating experiments were carried out using an 808 nm laser (MDL-N-808-10 W, Changchun, China). UV-Vis spectra were obtained with a UV-Vis spectrophotometer (YOKE T2602, Shanghai, China). Neutrophil membrane vesicles were extruded with Avanti mini extruder (610000, Avanti polar lipids, U.S.A.). Multichannel microplate reader (Thermo Scientific, Multiskan FC, U.S.A.) was employed to record the optical density (OD) of bacterial suspension. Bacterial fluorescence images were acquired with a confocal laser scanning microscope (CLSM, Zeiss, LSM880, Germany). Zeta potential and hydrodynamic size were measured with a zeta-sizer Nano-ZS (Malvern Instruments Inc. Worcestershire, UK). Glass transition temperature (T_g_) was determined using a differential scanning calorimetry (DSC) analyzer (DSC 4000, PerkinElmer, U.S.A.). The in vivo imaging experiments were performed by using IVIS imaging system (IVIS® Lumina III, PerkinElmer, U.S.A.).

### Preparation of PLGA nanoparticles loaded with ICG and RIF

PLGA nanoparticles co-loaded with ICG and RIF (NP-ICG/RIF) were fabricated *via* an O/W emulsion method. Briefly, PLGA (40 mg), ICG (5 mg) and RIF (1.6 mg) were dissolved in 500 µL dichloromethane (DCM). Then, the homogenous DCM solution was transferred to a 5 mL glass vial, followed by adding 1.3 mL of 0.5% PVA dissolved in water (w/v). Subsequently, the mixture was sonicated with a probe ultrasonicator at a power percentage of 15% (3 s on, 3 s off for 5 min) on top of ice. Another 0.6 mL of 0.5% PVA solution (w/v) was then added to homogenize the emulsion, which was stirred at room temperature for 4 h to remove DCM by evaporation. The obtained nanoparticles were washed several times with deionized water using an Amicon ultra-4 centrifugal filter to collect final NP-ICG/RIF products and the NP-ICG/RIF were stored in deionized water at 4℃ for future use. The loading content of ICG and RIF was studied with a UV-Vis spectrophotometer.

### Neutrophil-like cell membrane derivation

Neutrophil-like cell membrane was obtained following a previously published protocol [39]. Briefly, HL-60 cells were recovered from liquid nitrogen, followed by culturing the cells in IMEM medium supplemented with 20% fetal bovine serum and 1% penicillin & streptomycin for 4 days. To differentiate HL-60 cells, DMSO was added in the medium (1% v/v) and cells were cultured for 4 days. Afterwards, the cells were lysed through a lysing buffer containing 30 mM Tris-HCl (pH 7.5), 225 mM d-mannitol, 75 mM sucrose, 0.2 mM EDTA, a protease and phosphatase inhibitor cocktail, followed by homogenization using a Dounce homogenizer (150 passes) in ice bath. The homogenized suspension was then subjected to centrifugation at 10,000 g for 10 min at 4 °C to remove organelles. Thereafter, the supernatant was centrifuged at 20,000 g for 60 min at 4 °C to collect the cell membrane. The cell membrane was suspended in PBS solution, and extruded for 15 passes with 400 nm porous polycarbonate membranes by Avanti mini extruder to obtain neutrophil-like cell membrane vesicles (NMVs). Membrane concentration was determined by quantification of membrane surface protein using a BCA kit, and bovine serum albumin (BSA) was used as the standard.

### Preparation and characterization of NM-NP-ICG/RIF

To prepare NM-NP-ICG/RIF, NP-ICG/RIF was first mixed with HL-60 membrane at a nanoparticle-to-membrane protein weight ratio of 2:1 (0.5 mg mL^− 1^: 1 mg mL^− 1^). The mixture was coextruded for 15 passes with 400 nm porous polycarbonate membranes by Avanti mini extruder to prepare neutrophil-like cell membrane-coated nanoparticles.

To examine the morphology, nanoparticles were negatively stained with phosphotungstic acid (15 mg mL^− 1^) and examined by transmission electron microscopy (TEM). From TEM images, the thickness of membrane coating was estimated based on 11 nanoparticles and 11 different places. The hydrodynamic size and surface zeta potential were measured by Malvern zeta-sizer Nano-ZS. Rhodamine B (excitation/emission = 540/625 nm) and DiO (excitation/emission = 484/501 nm) were used to label nanoparticles and NMVs, respectively, for confocal microscopic observations and flow cytometry analysis.

The T_g_ of bulk PLGA materials and PLGA nanoparticles was measured by a DSC analyzer. Briefly, samples (6.2 mg) were placed onto a sealed aluminum plate and heated from − 10℃ to 80℃ at a heating rate of 20℃ min^-1^ under a nitrogen flow rate of 50 mL min^-1^. The obtained DSC curves were processed using Pyris software to determine the value of T_g_.

### Identification of membrane proteins

The membrane proteins presented on the nanoparticles and HUVECs were verified by SDS-PAGE and Western blot. In the SDS-PAGE analysis, NP (10 µg), NM (10 µg) and NM-NP (20 µg) were mixed with SDS loading buffer and loaded into each well of a 10% Tris/glycine SDS-polyacrylamide gelatin in an electrophoresis chamber system. The proteins were separated at 80 V for 0.5 h and then at 120 V for 1 h. Subsequently, the gel was stained with Coomassie brilliant blue for 1 h prior to protein imaging. For Western blot analysis, samples with equivalent amounts of proteins were firstly separated with SDS-PAGE. After that, the proteins were transferred to the nitrocellulose membranes, followed by the blockage with milk for 1 h. Then the blots were incubated with antibodies against β2 integrin, TRL4, CD14 and ICAM-1 at 4 ℃ overnight, and further incubation with horseradish peroxidase conjugated anti-mouse or anti-rabbit IgG at 25 ℃ for final visualization.

### Photothermal heating and photothermal responsive drug release

Photothermal heating property of the as-prepared nanoparticles was characterized with an 808 nm laser. Different nanoparticles (534 µg mL^− 1^, 500 µL) in PBS were added to 48-well plates, followed by the irradiation of laser for 5 min at a power density of 1.5 W cm^− 2^. The temperature was captured at different time points with an infrared thermometer. Besides, the heating properties of NM-NP-ICG/RIF at different power densities and different concentrations were also studied by following the same method as aforementioned.

Photothermal-responsive drug release of RIF was determined in PBS at 37 °C. NM-NP-ICG/RIF (10 mg mL^− 1^, 250 µL) were added to a 48-well plate and irradiated with laser at 1.5 W cm^− 2^ for 5 min. Then, the nanoparticles were centrifuged and the supernatants were collected to determine the amount of released drug at different time points over 24 h. The concentration of RIF was measured via UV-Vis spectra at wavelength of 474 nm. NP-ICG/RIF and NM-NP-ICG/RIF nanoparticles (10 mg mL^− 1^, 250 µL) without laser irradiation were used as controls. All experiments were performed in triplicates.

### In vitro targeting to inflammatory HUVEC cells

HUVEC cells were seeded into 12-well plates and cultured for 24 h. Then, LPS was added to DMEM medium (1 µg mL^− 1^) to stimulate the cells for 24 h. Afterwards, the cells were washed with PBS and incubated with DiO-labeled neutrophil cell membrane vesicles (NMVs) (50 µg mL^− 1^). The cells were then stained with Hoechst 33,342 and imaged under a fluorescent microscope equipped with green and blue filter. In order to compare the binding property of NM-NP-ICG/RIF and NP-ICG/RIF, Rhodamine B was loaded to label PLGA nanoparticles, while DiO was used to stain neutrophil-like cell membrane. For the quantitative characterization, cells were collected and analyzed with flow cytometry. The mean fluorescence intensity (MFI) was calculated using FlowJo software, all the MFI values represent the mean values of three replications.

### In vitro photothermal antibacterial tests

*P. aeruginosa* was inoculated into 10 mL of MHB medium and shaken at 37 °C for 18 h. Then, the bacterial cells were pelleted by centrifugation at 3500 g for 5 min at 4 °C and washed three times with PBS. The bacterial suspension in MHB was adjusted to a concentration of 10^8^ CFU mL^− 1^. Different nanoparticles with the same ICG content (15 µg mL^− 1^) were mixed with the bacterial suspension (200 µL) and irradiated with the laser at 1.5 W cm^− 2^ for 5 min. The viability of the bacterial cells was analyzed with plate counting assay, all experiments were run in three replicates.

In addition, BacLight live/dead fluorescent kit was employed to qualitatively characterize the viability of the treated bacterial cells. In brief, Syto 9 nucleic acid stain (1.5 µL) and propidium iodide (PI) (1.5 µL) were mixed and added to 1 mL of the treated bacterial solution, where green fluorescent Syto 9 stains live bacteria and red fluorescent PI stains dead bacteria. The mixture was then incubated in dark at 25℃ for 15 min. Finally, the stained bacterial solution (5 µL) was mounted on a glass slide and covered with a coverslip, followed by imaging on the confocal microscope.

### Endotoxin neutralization and anti-inflammation activities

LPS-FITC (0.1 µg mL^− 1^) and NP (1 mg mL^− 1^) or NM-NP (1 mg mL^− 1^) were co-incubated in dark for 30 min, followed by centrifugation and imaging with a fluorescence imaging system. To quantify the absorbed LPS, the precipitates were resuspended into PBS and analyzed with a fluorescence spectrophotometer. The absorbance was calculated against standard calibration curve to determine the concentration of LPS-FITC. A positive control group of LPS-FITC (0.1 µg mL^− 1^) without nanoparticle treatment was used to calculate the removal rate of LPS by NM-NP and NP. The data were collected as mean values of three replicates.

For the anti-inflammation treatment, LPS (0.1 µg mL^− 1^) and different samples (2 mg mL^− 1^) were co-incubated with RAW264.7 cells in DMEM medium for 24 h. Subsequently, the cells were stained with 10 µM of DCFH-DA and the images were acquired under an inverted fluorescent microscope (Zeiss, Germany) to evaluate the intracellular ROS level. The cell culture supernatant was collected and centrifuged at 20,000 g to remove all the nanoparticles, after which the supernatant was tested with IL-6 and TNF-α ELISA kits to determine the cytokine level. All the experiments were repeated for three times.

### Biocompatibility assessments

Alamar blue cytotoxicity assay and hemolysis assay were carried out to assess the biocompatibility of NM-NP-ICG/RIF. In Alamar blue cytotoxicity assay, HACAT, HUVEC and HEK 293T cells were cultured with RPMI 1640 or DMEM medium supplemented with 10% FBS in 96-well plates at a cell density of 5000 cells/well for 24 h. Afterwards, the cells were incubated with NM-NP-ICG/RIF at different concentrations (0, 178, 356, 534, 712, 1069 µg mL^− 1^) and cultured for another 24 h. The treated cells were then washed 3 times with PBS and Alamar blue reagent was subsequently added. After 5 h incubation at 37℃, the fluorescence intensity was recorded on a microplate reader at *λ*_ex_ of 530–560 nm and *λ*_em_ of 590 nm.

For the hemolytic assay, whole blood freshly collected from the heart of mouse was centrifuged at 2200 g for 5 min to pellet red blood cells (RBCs). After 3 times washing with PBS, the obtained RBCs were then resuspended in PBS and incubated with NM-NP-ICG/RIF at different concentrations (178, 356, 534, 712, 1069 µg mL^− 1^) for 1 h. PBS without the nanoparticles and Triton-X 100 (0.1%, v/v) were set as negative control and positive control, respectively. After centrifugation at 2200 g for 5 min, the absorbance of the supernatant at 576 nm was measured with a microplate reader. The hemolytic rate (%) was calculated as follows:

1 $$\mathrm{Hemolytic}\;\mathrm{rate}=\frac{{\mathrm{OD}}_{\mathrm s}-{\mathrm{OD}}_{\mathrm{nc}}}{{\mathrm{OD}}_{\mathrm{pc}}-{\mathrm{OD}}_{\mathrm{nc}}}\times100\%$$

where OD_s_, OD_nc_ and OD_pc_ represent the absorbance of samples, negative control and positive control, respectively. All the OD values represent the mean value of five replications.

### In vivo photothermal antibacterial efficacy evaluation on *P. aeruginosa* infection

All the in vivo experiments performed were approved by the Institutional Animal Care and Use Committee of School of Pharmaceutical Sciences (Shenzhen), Sun Yat-Sen university. The photothermal drug delivery system is more suitable for treatment of superficial and localized infections rather than deep infection or systemic infection. In addition, infection caused by *P. aeruginosa* through subcutaneous route is a common hospital-acquired infection [40]. In this study, a murine skin abscess *P. aeruginosa* infection model was established. Briefly, six-week-old male Balb/c mice (20–22 g) were first anesthetized with an intraperitoneal injection of 1% pentobarbital (200 µL), and followed by shaving with depilatory creams and disinfection with 70% ethanol. Then *P. aeruginosa* (1.5 × 10^10^ CFU mL^− 1^, 25 µL) was injected to the right rear back of mice.

To visualize the nanoparticles in the infected mice, the infected mice were randomly divided into two groups: control group treated with NP-ICG/RIF and experimental group treated with NM-NP-ICG/RIF (each group with 3 mice). After 12 h of infection, NM-NP-ICG/RIF (50 µL) or NP-ICG/RIF nanoparticles (50 µL) both containing ICG at 5 µg mL^− 1^ were injected to the infectious site. The fluorescence of ICG in the mice was imaged at time point of 0 h, 6 h, 12 and 24 h. The average radiant efficiency was evaluated by IVIS imaging system.

To evaluate the in vivo antibacterial activity of the nanoparticles, the mice were randomly divided into 6 groups for treatment, namely PBS, RIF (8 µg mL^-1^), NP-ICG/RIF (15 µg mL^-1^ of ICG, 8 µg mL^-1^ of RIF), NM-NP-ICG/RIF (15 µg mL^-1^ of ICG, 8 µg mL^-1^ of RIF), NP-ICG/RIF + NIR and NM-NP-ICG/RIF + NIR. A severe subcutaneous infection model in mice was established by injection of *P. aeruginosa* (1.5 × 10^10^ CFU mL^-1^, 25 µL) to the right rear back of mice. Different nanoparticles (50 µL) were subcutaneously injected into the infectious site 12 h post infection and followed by laser irradiation (808 nm, 1 W cm^-2^) for 5 min after 24 h of nanoparticle injection. Meanwhile, local temperature of the infection was monitored by an IR thermal camera. After laser irradiation for 4 h, the infectious tissues were collected and homogenized in 5 mL normal saline for *P. aeruginosa* colony counting. To further prove that NM-NP-ICG/RIF could neutralize LPS and reduce the bacterial LPS-induced inflammatory responses, cytokines (TNF-α, IL-6) were quantified in infectious tissue homogenate using commercial ELISA kits. The survival rate of the mice was recorded for 10 days.

### Statistical analysis

The experiment data were demonstrated as means ± standard deviation, where they were repeated at least three times. Statistical significance (*p* < 0.05) was evaluated by Student’s t-test and only two groups were compared. In all tests, the statistical significance for the tests was set at *p < 0.05, **p < 0.01, ***p < 0.001 and ****p < 0.0001.

## Results

### Nanoparticles preparation and characterization

**Fig. 1:**
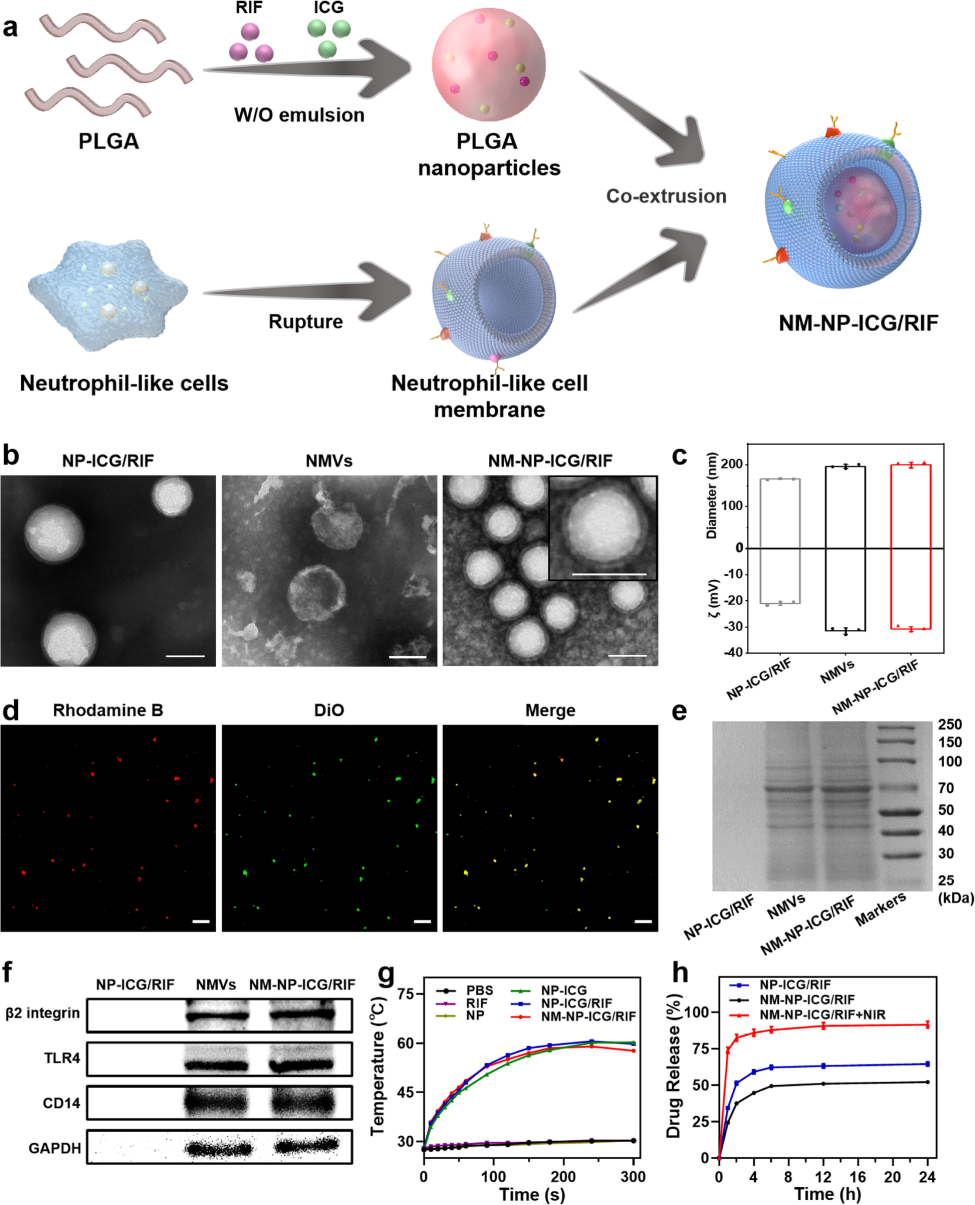
Preparation scheme and characterizations of the neutrophil-like cell membrane-coated nanoparticles. **a** NM-NP-ICG/RIF prepared by co-extrusion of neutrophil-like cell membrane and PLGA nanoparticles loaded with antibiotic rifampicin (RIF) and photothermal agent indocyanine green (ICG). **b** Transmission electron microscopic (TEM) images of NP-ICG/RIF, NMVs and NM-NP-ICG/RIF. All samples were negatively stained with phosphotungstic acid (Scale bar: 100 nm). **c** Hydrodynamic size and zeta-potential of NP-ICG/RIF, NMVs and NM-NP-ICG/RIF (*n* = 3). **d** Confocal laser scanning microscopic (CLSM) images of NM-NP-ICG/RIF with DiO-labeled NMVs (green) and Rhodamine B-labeled NP-ICG/RIF (red) (Scale bar :10 μm). **e** Sodium dodecyl sulfate polyacrylamide gel electrophoresis (SDS-PAGE) analysis of proteins presented on NP-ICG/RIF, NMVs and NM-NP-ICG/RIF. **f** Western blot analysis of β2 Integrin, TLR4 and CD14 on NMVs and NM-NP-ICG/RIF. **g** Temperature change of PBS, PLGA NP, RIF and various ICG-loaded nanoparticles (534 µg mL^− 1^) irradiated with an 808 nm laser (1.5 W cm ^− 2^) for 5 min (*n* = 3). **h** In vitro release of rifampicin from the NP-ICG/RIF, NM-NP-ICG/RIF and NM-NP-ICG/RIF after laser irradiation for 5 min (*n* = 3)

Neutrophil-like cell membrane coated nanoparticles were prepared through a two-step process (Fig. 1a). Firstly, PLGA nanoparticles co-loaded with ICG and RIF (NP-ICG/RIF) were fabricated using a typical O/W emulsion method. Then, neutrophil-like cell membrane vesicles (NMVs) derived from HL-60 cells were co-extruded with the NP-ICG/RIF to obtain NMVs-coated NP-ICG/RIF (NM-NP-ICG/RIF). UV-Vis spectra of NP-ICG/RIF showed characteristic absorption peaks of both RIF (474 nm) and ICG (794 nm) (Figure S1), indicating the successful loading of these two drugs in PLGA nanoparticles. The loading content of ICG and RIF were further determined to be 3.51% ± 0.23% and 1.99% ± 0.15%, respectively (Table S1). After co-extrusion of NP-ICG/RIF and NMVs, the nanoparticles exhibited a membrane layer with an average thickness of 8.5 ± 1.0 nm coated on the cores (Fig. 1b). This finding is in agreement with other membrane-coated nanoparticles in the literature [41, 42]. The increase of nanoparticle size from 166 to 200 nm and decrease of zeta potential from − 21 mV to -31 mV further confirmed the presence of cell membrane coated on the surface of NP-ICG/RIF (Fig. 1c). The polydispersity of NM-NP-ICG/RIF was determined to be 0.164, indicating that the membrane-coated nanoparticles are homogeneous (Table S2). To verify the coating efficiency of the nanoparticles, fluorescent dyes DiO and Rhodamine B were used to label NMVs and NP-ICG/RIF, respectively. The overlap (yellow) of fluorescent signals of DiO (green) in NMVs and Rhodamine B (red) in NP-ICG/RIF demonstrated the high coating efficiency of the nanoparticles (Fig. 1d). More importantly, the NM-NP-ICG/RIF inherited the proteins of NMVs (Fig. 1E), and the important proteins that are responsible for inflammation-targeting and LPS-binding including β2 integrin, TRL4 and CD14 [43–45] were indeed observed in the western blotting images of NM-NP-ICG/RIF (Fig. 1f).

As the co-loading of photothermal reagent ICG and antibiotic RIF in the NM-NP-ICG/RIF was aimed to achieve photothermal antibacterial function and photothermal-triggered antibiotic release, the photothermal heating property and release profile of rifampicin were investigated. All the ICG-loaded nanoparticles, including NP-ICG, NP-ICG/RIF and NM-NP-ICG/RIF, were heated up to 58 ℃ within 5 min upon the irradiation of near-infrared (NIR) laser 808 nm at 1.5 W cm^− 2^ (Fig. 1g), while the laser irradiation with the same conditions almost showed no heating effect on phosphate-buffered saline (PBS), RIF or PLGA NP alone. Further investigation of the photothermal properties of NM-NP-ICG/RIF revealed that the heating temperature was both laser power-dependent and concentration-dependent (Figure S2a and S2b). Since the glass transition temperature (T_g_) of NP-ICG/RIF nanoparticles is 35.79 °C (Figure S3), the photothermal heating temperature above the T_g_ upon NIR irradiation would lead to glass transition and flexible rubbery state of PLGA nanoparticles. Therefore, the nanoparticles would change from a rigid solid state to an elastic or viscous solid state, resulting in the collapse of the nanoparticles and thus accelerating the release of rifampicin from NM-NP-ICG/RIF [38]. To verify this hypothesis, the morphology of the nanoparticles after NIR irradiation was analyzed (Figure S4). Indeed, the nanoparticles collapsed and changed from spherical particles to amorphous clusters. The photothermal drug release profile is shown in Fig. 1h, compared with the NM-NP-ICG/RIF without laser irradiation, the amount of RIF released increased from 52.15% ± 1.06 to 91.55% ± 2.37% in 24 h after laser irradiation. To investigate if the fast drug release profile is due to the elevated temperature, drug release from NM-NP-ICG/RIF nanoparticles was studied at different temperatures in dark (Figure S5). More than 75% of the drug were released at 58 ℃ in a 24 h time interval, compared to around 40% and 30% drug release at 37 ℃ and 25 ℃, respectively. This result indicates that the NIR irradiation could lead to photothermal deformation of NM-NP-ICG/RIF and thus trigger fast release of the encapsulated drug.

### In Vitro Targeting of NMVs-coated nanoparticles to inflammatory vascular endothelial cells

**Fig. 2:**
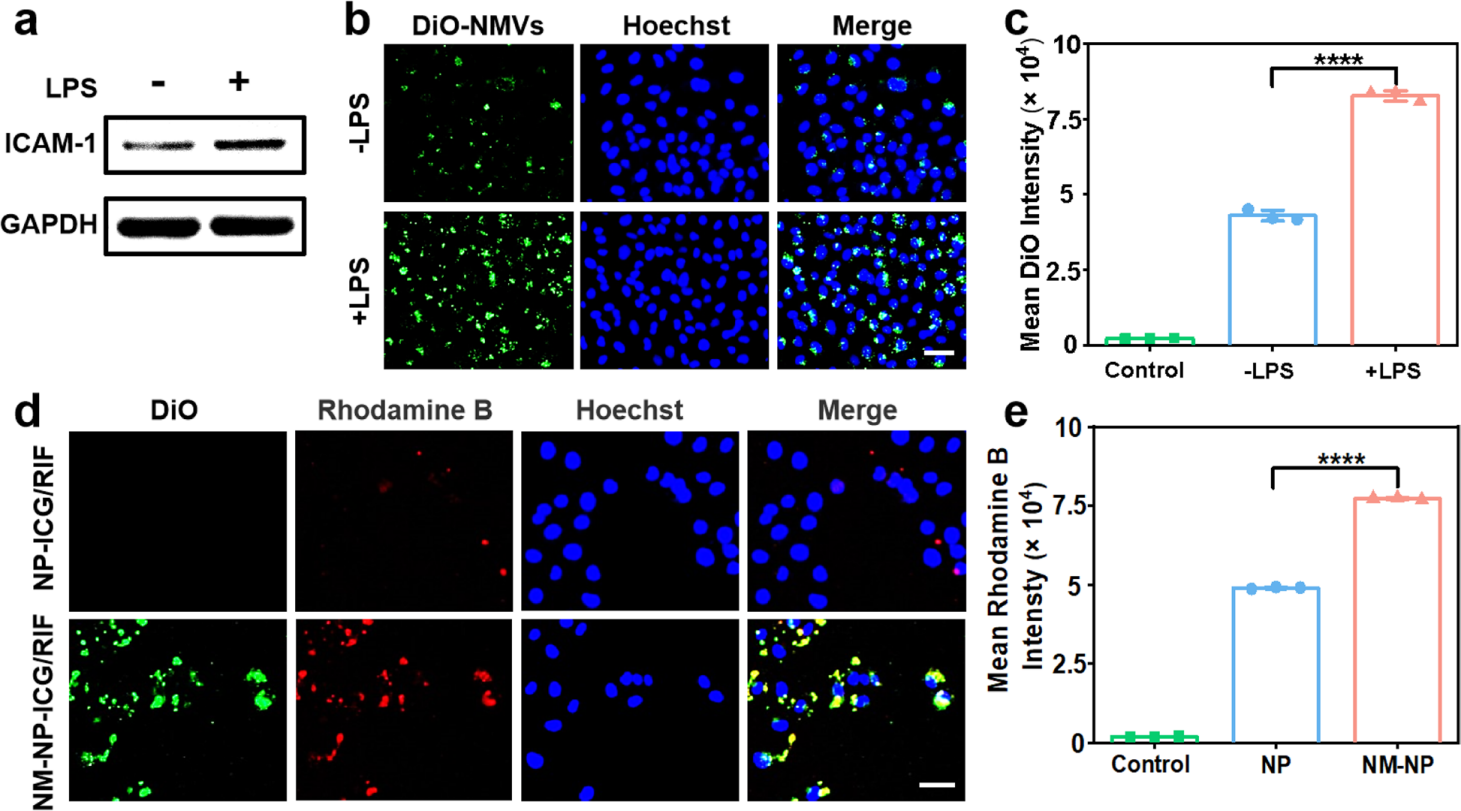
In vitro targeting of NMVs and NM-NP-ICG/RIF to inflammatory vascular endothelial cells. **a** Western blot analysis of ICAM-1 in normal human umbilical vein endothelial cells (HUVECs) and LPS-stimulated HUVECs. **b** CLSM images of normal and LPS-stimulated HUVECs after incubation with NMVs (50 µg mL^− 1^) at 37 °C for 2 h. (Green: DiO-labelled NMs; blue: Hoechst-labeled nuclei. Scale bar, 50 μm.). **c** Mean fluorescence intensity of DiO in normal and LPS-stimulated HUVECs characterized by flow cytometry (*n* = 3). **d** CLSM images of LPS-stimulated HUVECs after incubation with NP-ICG/RIF (50 µg mL^− 1^) and NM-NP-ICG/RIF (50 µg mL^− 1^) at 37 °C for 2 h. (Green: DiO-labeled NMVs; red: Rhodamine B-labeled NP-ICG/RIF; blue: Hoechst-labeled nuclei. Scale bar: 50 μm). **e** The mean fluorescence intensity of Rhodamine B in HUVECs after incubation with the nanoparticles with or without NMVs coating (*n* = 3). (****p < 0.0001, Student’s t-test)

The interaction of β2 integrin expressed on neutrophil and ICAM-1 (intercellular cell adhesion molecule-1) presented on inflammatory vascular endothelial cells plays an important role in adhesion and transmigration of neutrophil to the bacterial infection site [46, 47]. As β2 integrin was presented on neutrophil-like cell membrane-coated nanoparticles (Fig. 1f), these nanoparticles were presumed to specifically target inflammatory endothelium. ICAM-1 was found to be present on human umbilical vein endothelial cells (HUVECs) and LPS enhanced the expression of ICAM-1 (Fig. 2a). Then, the binding capability of NMVs to inflamed HUVECs was investigated. The fluorescent dye DiO-labeled NMVs were incubated with HUVECs with and without LPS. The LPS-stimulated cells exhibited significantly higher green fluorescence as compared to the non-stimulated cells (Fig. 2b and S6a), suggesting the strong specific adhesion of NMVs to inflammatory endothelial cells. The higher fluorescence was further confirmed with quantification analysis of DiO fluorescence intensity through flow cytometry (Fig. 2c and S6a). Subsequently, the binding capability of NM-NP-ICG/RIF to LPS-stimulated HUVECs was studied, where DiO was used to label NMVs and Rhodamine B employed to label NP-ICG/RIF. CLSM images showed that the cells incubated with NM-NP-ICG/RIF displayed obvious green (DiO) and red (Rhodamine B) fluorescence, while cells treated with NP-ICG/RIF showed much weaker red fluorescence (Fig. 2d and S6b). Flow cytometry analysis further confirmed that the mean fluorescence intensity of the Rhodamine B in the NM-NP-ICG/RIF-treated cells is 43.94% ± 0.48% higher than that of NP-ICG/RIF (Fig. 2e and S6b).

### In vitro antibacterial activities

**Fig. 3:**
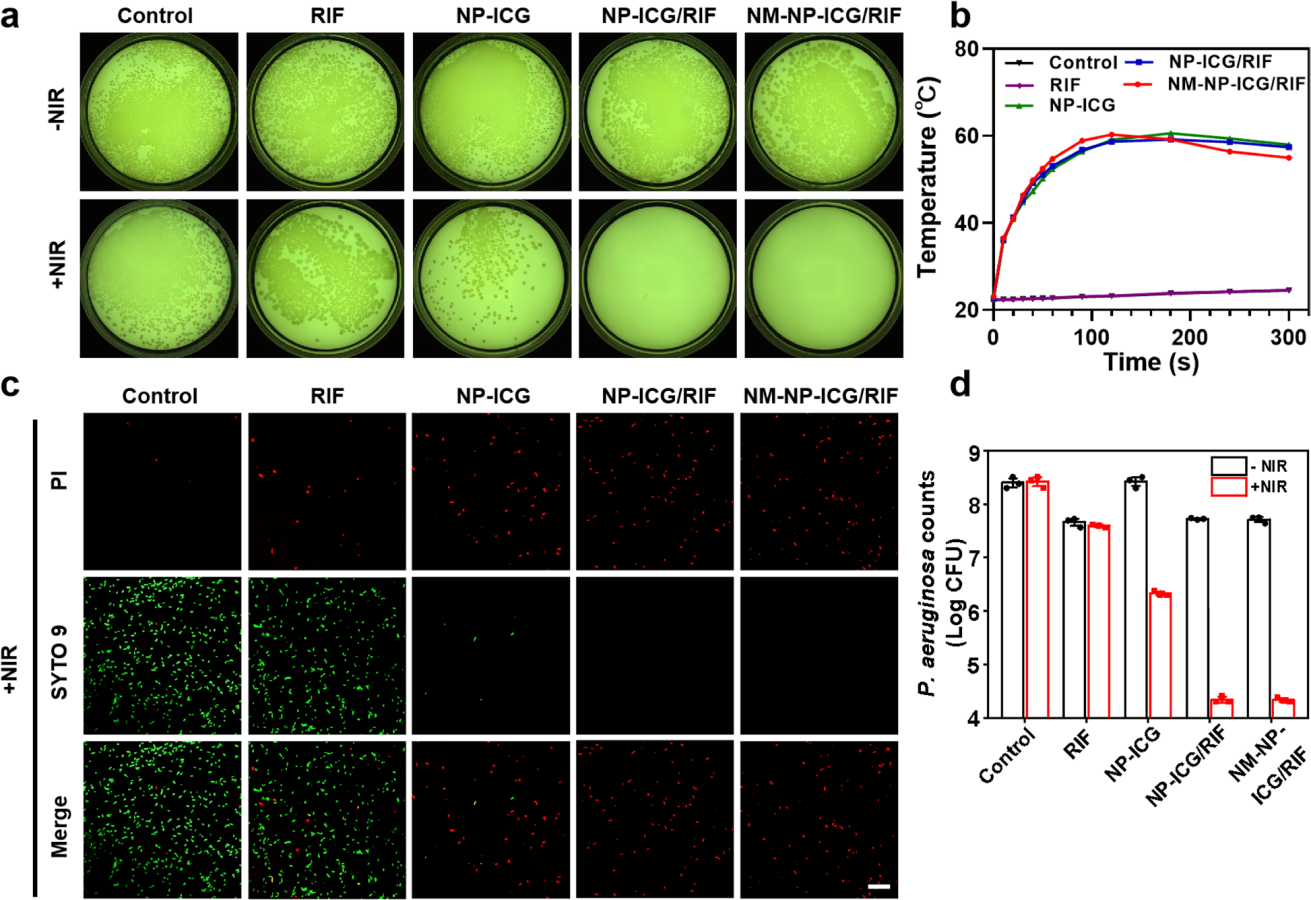
In vitro synergistic photothermal antibacterial activity of nanoparticles containing both RIF and ICG. **a** Colony photos of *P. aeruginosa* upon treatment with RIF (8 µg mL^− 1^), NP-ICG (15 µg mL^− 1^ of ICG), NP-ICG/RIF and NM-NP-ICG/RIF (containing RIF: 8 µg mL^− 1^, ICG: 15 µg mL^− 1^) under NIR laser irradiation (808 nm, 1.5 W cm^− 2^) for 5 min or no NIR laser irradiation (initial bacterial loading: ~10^8^ CFU mL^− 1^). **b** Photothermal heating curves of different samples incubated with *P. aeruginosa* under NIR laser irradiation (808 nm, 1.5 W cm^− 2^) for different time periods (*n* = 3). **c** CLSM images of *P. aeruginosa* under the same treatment conditions as aforementioned in Fig. 3A, and followed by staining with LIVE/DEAD BacLight bacterial viability kit where green fluorescent dye stains all the bacterial cells and red PI dye stains bacterial cells with damaged membrane (Scale bar: 20 μm). **d** The counts of *P. aeruginosa* colonies under the same treatment conditions as aforementioned in Fig. 3a (*n* = 3)

With success in using NMVs-coated nanoparticles to target inflammatory vascular endothelial cells, photothermal reagent ICG and antibiotic RIF were encapsulated in the neutrophil-like cell membrane-coated nanoparticles to achieve synergistic antibacterial activity. Antibiotic RIF (concentration: 8 µg mL^− 1^) and ICG-loaded nanoparticles (NP-ICG) (15 µg mL^− 1^ of ICG ) used alone displayed negligible antibacterial activity against *P. aeruginosa* (Fig. 3a). Although the NIR laser irradiation could improve the antibacterial activity of NP-ICG by photothermally heating the nanoparticles to ~ 58 °C in 5 min, the antibacterial efficacy was not sufficiently high enough to eradicate *P. aeruginosa* (Fig. 3a and b). However, the nanoparticles containing both ICG and RIF (NP-ICG/RIF and NM-NP-ICG/RIF) under NIR irradiation exhibited potent antibacterial activity with complete eradication of *P. aeruginosa* (Fig. 3a). The antibacterial potency was due to the synergistic effect of photothermal activity of ICG and bactericidal activity of RIF released from nanoparticles upon photothermal triggering. In addition, the photothermal treatment of NP-ICG under NIR irradiation could lead to bacterial membrane disruption, making bacteria permeable to red PI dye (Fig. 3c). The enhanced permeability of bacterial membrane to RIF might also contribute to the synergistic antibacterial activity. The live/dead fluorescence staining and bacterial quantification analysis both further confirmed the superior antibacterial activity of NP-ICG/RIF and NM-NP-ICG/RIF under NIR irradiation over mono-treatment with RIF or NP-ICG (4.1-log, 4.1-log, 0.8-log and 2.1-log reduction for NP-ICG/RIF, NM-NP-ICG/RIF, RIF and NP-ICG, respectively) (Fig. 3c and d and S7). Notably, NP-ICG/RIF and NM-NP-ICG/RIF with NIR irradiation achieved ~ 99.99% killing efficacy (4-log reduction) of *P. aeruginosa* in 5 min even with the initial bacterial loading of 10^8^ CFU mL^− 1^.

### Endotoxin neutralization and anti-inflammation activities of NMVs-coated nanoparticles

**Fig. 4:**
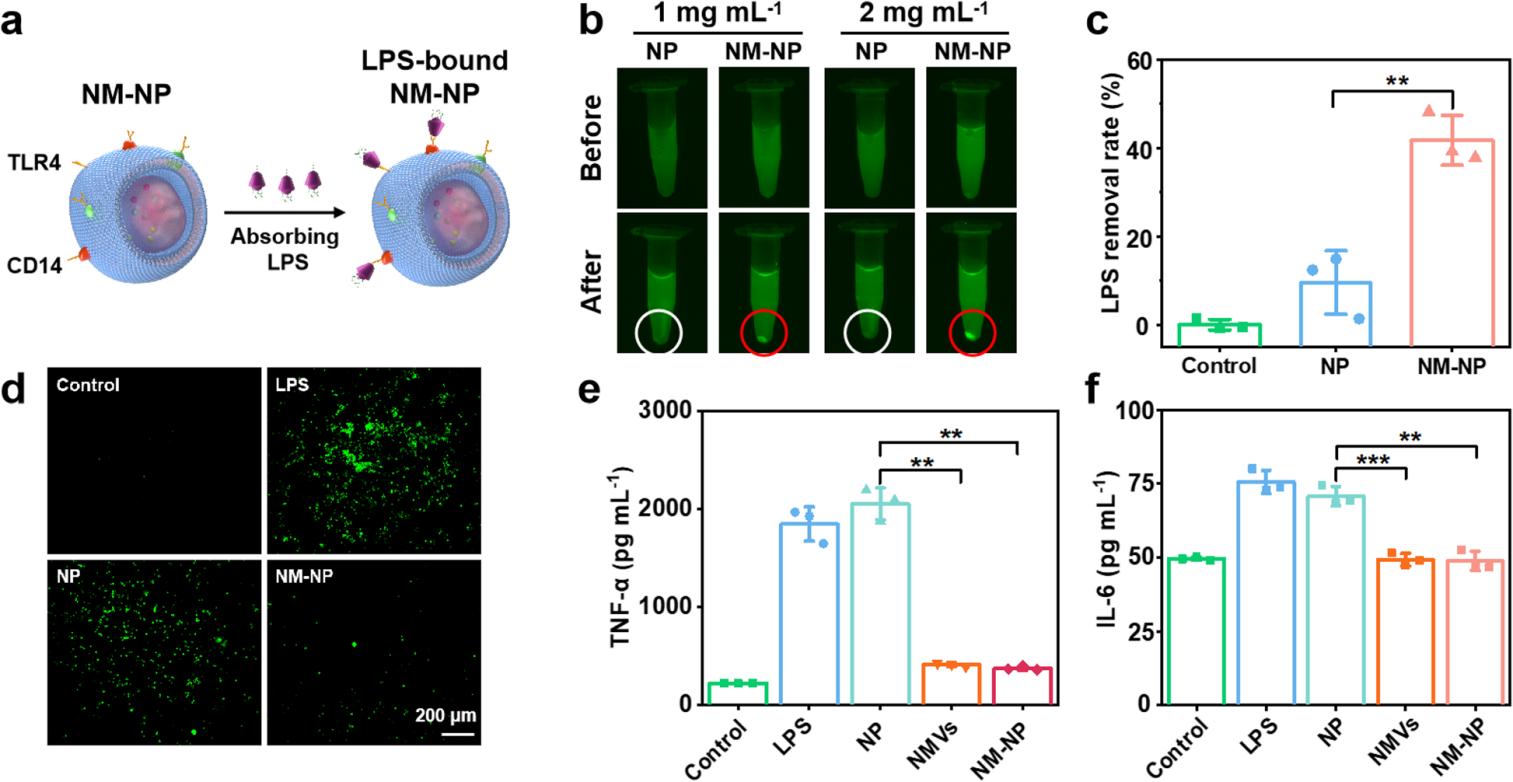
Endotoxin (LPS) neutralization and anti-inflammation activities of NMVs-coated nanoparticles (NM-NP). **a** Schematic illustration of the mechanisms for LPS neutralization and subsequent anti-inflammation of NM-NP. **b** Fluorescent images of LPS-FITC solution (0.1 µg mL^− 1^) before and after incubation and centrifugation with NP or NM-NP (1 or 2 mg mL^− 1^). **c** Binding efficiency of NP and NM-NP to LPS-FITC (*n* = 3); **d** Intracellular ROS fluorescent images of untreated RAW264.7 macrophages, LPS-treated macrophages, and LPS-stimulated macrophages treated with blank NP or NM-NP. The amount of (**e**) TNF-α and (**f**) IL-6 secreted from macrophages under different treatment (*n* = 3, **p < 0.01 and ***p < 0.001, Student’s t-test)

Since TLR4 and CD14 presented on neutrophil-like cell membranes can bind to LPS (endotoxin), we hyposized that NMVs-coated nanoparticles were able to neutralize LPS and alleviate LPS-induced inflammatory responses (Fig. 4a). To evaluate the endotoxin neutralization capability of neutrophil-like cell membrane NMVs-coated nanoparticles, FITC-tagged LPS (LPS-FITC) was co-incubated with NM-NP for 30 min, and followed by centrifugation to spin down the nanoparticles. The obtained precipitates displayed obvious green fluorescence of FITC (red circle in Fig. 4b), while no fluorescence was found in the precipitates of NP without membrane coating (white circle in Fig. 4b), suggesting that NMVs-coated nanoparticles can effectively bind to LPS. Moreover, the binding efficacy was concentration-dependent, and more LPS could be bound when the concentration of NM-NP increased from 1 mg mL^− 1^ to 2 mg mL^− 1^. The quantification results of the binding efficicency showed that NM-NP (1 mg mL^− 1^) removed nearly 40% of the total LPS, significantly higher than 10% LPS removal of NP without membrane coating (Fig. 4c). When the concentration of NM-NP increased to 2 mg mL^− 1^, its LPS binding capability was further enhanced with 70% LPS removal rate (Figure S8). In view of the fascinating LPS-binding capability, NMVs-coated nanoparticles were expected to reduce LPS-triggered inflammatory responses. To prove this hypothesis, LPS and NMVs-coated nanoparticles were co-incubated with RAW264.7 macrophages for 24 h, and followed by the characterization of cell inflammatory responses. Firstly, intracelluar ROS, known as an indicator of inflammatory reactions, [48, 49] was analyzed with green fluorescent probe DCFH-DA. As shown in Fig. 4d, LPS triggered significant generation of intracellur ROS with obvious green fluorescence in macrophages, indicating severe inflammatory reactions. However, when the macrophages were co-incubated with NM-NP, green fluorescence was remarkably diminished, while the macrophages treated with the NP without neutrophil-like cell membrane coating still exhibited prominent green fluorescence. This observation indicates the strong anti-inflammatory activity of NMVs. Inflammatory cytokines TNF-α and IL-6 in the cell culture medium were then quantified using the corresponding Enzyme-Linked Immunosorbant Assay (ELISA) kits (Fig. 4e and f). NMVs and NM-NP significantly reduced the secretion of TNF-α and IL-6 by macrophages as compared with NP and LPS control groups.

### Biocompatibility assessments

To verify the biocompatibility of the NM-NP-ICG/RIF nanoparticles, cytotoxicity of the nanoparticles towards HUVECs, HaCaT cells (a human skin keratinocyte line) and HEK 293T cells (a human embryonic kidney cell line) was evaluated, and hemolysis tests were carried out. NM-NP-ICG/RIF nanoparticles showed excellent biocompatibility with cell viability of higher than 84% even with the concentration of up to 1 mg mL^− 1^ (Figure S9a). These nanoparticles also exhibited negligible hemolysis at the concentration of up to 1 mg mL^− 1^ (Figure S9b). As the concentration of NM-NP-ICG/RIF we used for antibacterial tests was only 0.534 mg mL^− 1^, which was significantly lower than the highest concentration used for biocompatibility tests.

### In vivo targeted antibacterial activity

**Fig. 5:**
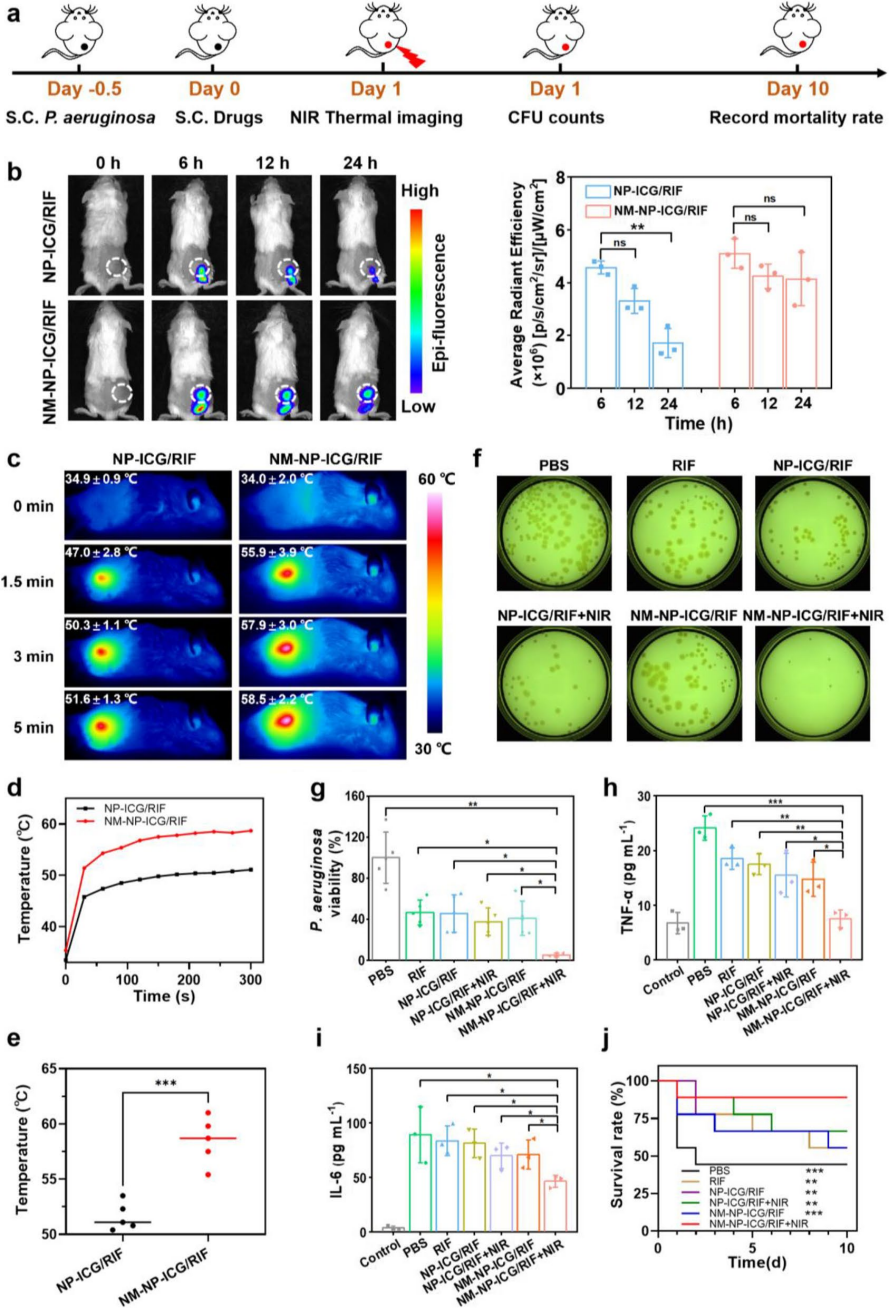
In vivo antibacterial activity against a *P. aeruginosa* murine skin abscess infection model. **a** In vivo antibacterial experimental time line. **b** In vivo fluorescence imaging (left) and quantitative analysis (right) of *P. aeruginosa*-infected mice after subcutaneous injection of NP- ICG/RIF or NM-NP-ICG/RIF (ICG concentration: 5 µg mL^− 1^) at different time intervals (The white circles indicate the *P. aeruginosa*-infected areas, *n* = 3). **c** Photothermal heating images of bacterial infection treated with NP-ICG/RIF or NM-NP-ICG/RIF (15 µg mL^− 1^ of ICG) under NIR irradiation (808 nm, 1.0 W cm^− 2^) for different time periods. (**d**) Photothermal heating curves and (**e**) Final temperatures reached after photothermal treatment with NP-ICG/RIF or NM-NP-ICG/RIF (ICG concentration: 15 µg mL^− 1^, *n* = 5). **f** Plate colony images and (**g**) quantification results of the bacteria obtained from infected tissues treated under different conditions (*n* = 5). Quantification of (**h**) TNF-α and (**i**) IL-6 in the infected tissues after various treatment (*n* = 3). **j** Survival rate of infected mice in 10 days after treatment under different conditions (*n* = 9). (*p < 0.05, **p < 0.01 and ***p < 0.001, Student’s t-test)

To investigate in vivo targeted antibacterial activity of NM-NP-ICG/RIF, a murine skin abscess infection model was established by subcutaneous injection of *P. aeruginosa* to the back of male Balb/c mice, [50, 51] followed by the subcutaneous injection of NM-NP-ICG/RIF to the infectious site (Fig. 5a). The dissipation of ICG fluorescence was recorded over a time span of 24 h after injection of NM-NP-ICG/RIF or NP-ICG/RIF (Fig. 5b). The fluorescence of NP-ICG/RIF and NM-NP-ICG/RIF both peaked at 6 h. However, the fluorescence of NP-ICG/RIF decreased dramatically at the time point of 12 and 24 h, with very weak fluorescence shown after 24 h. In contrast, the mice treated with NM-NP-ICG/RIF still showed significant fluorescence even after 24 h. The fluorescence intensity of NM-NP-ICG/RIF was more than twice as high as that of NP-ICG/RIF, indicating that the binding of NM-NP-ICG/RIF to inflammatory vascular endothelial cells prolonged retention time at the infection site.

Due to the targeted binding to the infection site, NM-NP-ICG/RIF generated more heat and led to a higher local temperature under the NIR irradiation when compared with NP-ICG/RIF (Fig. 5c). Photothermal heating curve (Fig. 5d) and final temperature record (Fig. 5e) showed that the coating with NMVs resulted in 7℃ higher heating temperature on average. After photothermal treatment, the infected tissues were harvested and homogenized for the counting of bacteria. As shown in Fig. 5f, g and a single treatment with NM-NP-ICG/RIF + NIR dramatically reduced the bacterial loading in the infectious wound. Over 95% bacteria were killed upon the treatment with NM-NP-ICG/RIF + NIR, and the antibacterial efficacy was significantly higher than other treatment groups. The results also testified that the synergistic antibacterial effect of photothermal reagent ICG and antibiotic RIF led to strong in vivo antibacterial activity. Moreover, the inflammatory cytokines level of TNF-α and IL-6 (Fig. 5h and i) in the bacteria-infected mice after treatment with NM-NP-ICG/RIF + NIR showed the neutrophil-like cell membrane coating significantly reduced the LPS-induced inflammatory responses in vivo. Thus, NM-NP-ICG/RIF + NIR with efficient bacterial eradication and LPS neutralization functions significantly improved the survival rate of infected mice (Fig. 5j). More than a half of infected mice died within 10 days after infection. Although the single treatment with RIF, NP-ICG/RIF, NM-NP-ICG/RIF or NP-ICG/RIF under NIR irradiation could slightly increase the survival rate to 55.6% or 66.7%, the single treatment with NM-NP-ICG/RIF and NIR irradiation showed the highest therapeutic efficacy with survival rate improved to 88.9%, indicating the strong in vivo photothermal antibacterial activity of NM-NP-ICG/RIF against *P. aeruginosa* bacterial infection.

## Discussion

Neutrophils play an important role in immune defenses, particularly in the initial phase of inflammation [52]. Various biomaterials have taken advantages of inflammation-targeting functions of neutrophils to treat inflammation-related diseases such as rheumatoid arthritis, ischemia brain damage and cancers [23, 29, 30, 52]. However, the neutrophil-based biomaterials or drug delivery systems for treatment of infectious diseases have been rarely reported [53]. Although a very recent report showed that neutrophil membrane vesicles (NMVs) are able to encapsulate the antibiotic ceftazidime, the inflammation-targeting functions in vivo was not studied and antibacterial efficacy of ceftazidime-loaded NMVs was very limited (< 50% bacterial reduction in a bacterium-induced peritonitis model) [54]. The off-targeted release of antibiotic and unsatisfactory targeting capability of NMVs could be responsible for the weak potency. In this study, a photothermo-responsive antibacterial delivery system coated with neutrophil membranes (NM-NP-ICG/RIF) was developed to achieve enhanced antibacterial activity. On the one hand, the thermo-responsive PLGA core loaded with the photothermal agent ICG reduced the passive release of RIF from NM-NP-ICG/RIF, which was proven by the increased release of the antibiotic RIF from 52.15% ± 1.06 to 91.55% ± 2.37% in 24 h after laser irradiation (Fig. 1h). The photothermo-responsive release profile is beneficial for the reduction of off-target drug release of antibiotic. On the other hand, neutrophil membrane was coated on the surface of NP-ICG/RIF to further enhance the accumulation of nanoparticles in the microenvironment of bacterial infection. In vitro and in vivo experiments revealed that the NM-NP-ICG/RIF specifically bound to inflamed vascular endothelial cells (Fig. 2) and prolonged the retention time of nanoparticles in the infection site (Fig. 5b). The photothermo-responsive core and inflammation site-specific shell of NM-NP-ICG/RIF together contributed to the targeted delivery of antibacterial agents, resulting in high therapeutic efficacy.

Given the rising resistance of Gram-negative bacteria to the existing antibiotics, potentiation of antibiotics that are clinically used to treat Gram-negative bacteria could be a promising solution [55]. The key barrier for the repurposing of antibiotics is how to cross the double membrane structure and enhance the cellular uptake of antibiotic by Gram-negative bacteria. The photothermal activity of ICG loaded in nanoparticles damaged the bacterial membrane (Fig. 3c), and the disrupted bacterial membrane might lead to increased cellular uptake of antibiotic rifampicin by *P. aeruginosa*, resulting in potent antibacterial activity both in vitro (Fig. 3) and in vivo (Fig. 5). Therefore, this study proposed a novel strategy to repurpose the Gram-positive bacteria-specific antibiotic rifampicin that is difficult to penetrate through the outer membrane of Gram-negative bacteria, as effective antibiotic to combat Gram-negative bacterial infection.

Current antibacterial strategies against Gram-negative bacterial infections are mainly focused on killing the bacterial pathogens, but a major virulence of such infection, endotoxin LPS, has been neglected. The release of LPS from Gram-negative bacteria can cause serious consequences, such as inflammatory cytokine storms and subsequent sepsis [11–13]. Therefore, it is necessary to neutralize the endotoxins when treating bacterial infections. Although Zhang’s group reported that the nanoparticles coated with macrophage membrane was capable of neutralizing endotoxin and sequestering proinflammatory cytokines, [56] whether neutrophil-derived membrane can neutralize endotoxin and its mechanism have not been explored yet. In this study, we, for the first time, demonstrated that neutrophil membrane-coated nanoparticles neutrolized endotoxin (Fig. 4 and Figure S8) and reduced the endotoxin-induced cell inflammatory responses in vitro. The endotoxin neutralization property of the nanoparticles could be because the neutrophil membrane receptors expressed on the surface of nanoparticles, such as Toll-like receptor-4 (TLR4) and CD14 (Fig. 1f), exhibit specific affinity to LPS [33, 34].

In addition to potent antibacterial activity and endotoxin neutralization, the in vitro biocompatibility of NM-NP-ICG/RIF was also proven with no significant toxicity to HUVECs and HEK 293T cells (Figure S9). As the NM-NP-ICG/RIF is mainly composed of biocompatible PLGA (FDA-approved polymer for drug delivery), FDA-approved drugs RIF and ICG, its biosafety in vivo is expected, and the nanoparticles may thus be used for treatment of bacterial infections.

## Conclusions

We have successfully synthesized a neutrophil-like cell membrane-coated photothermal-responsive drug delivery system for targeted antibacterial and anti-virulence treatment of *P. aeruginosa* infections. The biocompatible and biodegradable PLGA nanoparticles loaded with both ICG and RIF generated significant heat and subsequently released RIF under the NIR irradiation, showing a synergistic antibacterial effect. Neutrophil-like cell membrane coating enabled the strong adhesion of nanoparticles to LPS-stimulated inflammatory cells, rendering enhanced and targeted photothermal therapy of bacterial infections. Importantly, the neutrophil-like cell membrane-coated nanoparticles bound to LPS effectively due to the high affinity of NMVs to LPS, reducing inflammatory response. In vivo studies on a murine skin abscess infection model further demonstrated the infection-targeting and LPS-neutralization activities, and the superior therapeutic efficacy of NM-NP-ICG/RIF. It is envisioned that the neutrophil-coated drug delivery system may be used to treat notorious multidrug-resistant bacterial infections.

## Supplementary Information

**Additional file 1:** **Figure S1.**UV-Vis spectra of different samples. **Figure S2.** Photothermal heatingcurves of NM-NP-ICG/RIF. (a) Photothermal heating curves of NM-NP-ICG/RIF (534 μg mL^-1^) under different NIR irradiation (808 nm) power densities. (b) Photothermal heating curves of NM-NP-ICG/RIF with different concentrationsunder NIR irradiation (808 nm, 1.5 W cm^-2^). **Figure S3**. DSC thermogramsof PLGA and NP-ICG/RIF nanoparticles. **Figure S4.** TEMimages of (a) NP-ICG/RIF and (b) NM-NP-ICG/RIF nanoparticles after photothermalheating. Scale bar: 200 nm. **Figure S5**. Rifampicin release profiles fromNM-NP-ICG/RIF at different temperatures in dark. **Figure S6.** *In vitro* binding of NM-NP-ICG/RIF to inflammatory HUVEC cells. (a) CLSM images (left) and flow cytometry analysis (right) of the binding of NMVs (50 μg mL^-1^) to HUVEC cells with or without LPS stimulation. (b) CLSM images (left) and flowcytometry analysis (right) of the binding of nanoparticles with or without NMVscoating to LPS-stimulated HUVEC cells. Scale bar: 200 μm. **Figure S7. **CLSM images of *P. aeruginosa* treated with control, RIF (8 μg mL^-1^), NP-ICG (15 μg mL^-1^ of ICG), NP-ICG/RIF (8 μg mL^-1^ of RIF, 15 μg mL^-1^ of ICG) and NM-NP-ICG/RIF (8 μg mL^-1^ of RIF,15 μg mL^-1^ of ICG) without laser, and stained with LIVE/DEADBacLight bacterial viability kit. Scale bar: 20 µm. **Figure S8.** Binding efficiency of NM-NP to LPS under differentconcentrations. (*n* = 3). **Figure S9.** Biocompatibility assessments of NM-NP-ICG/RIF. (a) Viability of HUVECs, HACAT and HEK 293T cells after incubation with NM-NP-ICG/RIF nanoparticles at different concentrations for 24 h. (*n*= 3). (b) Hemolysis of mouse red blood cells upon treatment with NM-NP-ICG/RIF nanoparticles at different concentrations. The PBS-treated group and 0.1% triton-treated group were used as negative and positive controls, respectively. (*n* = 3). **Table S1.** Loading content of ICG and RIF in different nanoparticles. **Table S2. **Polydispersity index (PDI) of different nanoparticles.

## Data Availability

The datasets during and/or analysed during the current study available from the corresponding author on reasonable request.

## Abbreviations

LPS: Lipopolysaccharide
TLR4: Toll-like receptor-4
ICG: Indocyanine green
RIF: Rifampicin
PLGA: Poly (lactic-co-glycolic acid)
NP-ICG/RIF: Nanoparticles loaded with both indocyanine green and rifampicin
NM-NP-ICG/RIF: Neutrophil membrane-coated nanoparticles loaded with both indocyanine green and rifampicin
NMVs: Neutrophil-like cell membrane vesicles
FDA: Food and Drug Administration
WHO: World Healthcare Organization
PTT: Photothermal therapy
PDT: Photodynamic therapy
NP: Nanoparticles
OD: Optical density
CLSM: Confocal laser scanning microscope
DSC: Differential scanning calorimetry
Tg: glass transition temperature
O/W: Oil in water
DCM: Dichloromethane
PVA: Polyvinyl alcohol
DMSO: Dimethylsulfoxide
EDTA: Ethylene diamine tetraacetic acid
BSA: Bovine serum albumin
TEM: Transmission electron microscopy
HUVECs: Human umbilical vein endothelial cells
SDS-PAGE: Sodium dodecyl sulfate polyacrylamide gel electrophoresis
MFI: Fluorescence intensity
MHB: Mueller Hinton broth
DCFH-DA: 2’,7’-dichlorodihydrofluorescein diacetate
FITC: Fluorescein isothiocyanate
ELISA: Enzyme-linked immuno sorbent assay
DMEM: Dulbecco’s modification of Eagle’s medium
RBCs: Red blood cells
PBS: Phosphate buffer saline
CFU: Colony forming unit
TNF-α: Tumor necrosis factorα
IL-6: Interleukin-6
Fig: Figure
NIR: Near-infrared
ICAM-1: Intercellular cell adhesion molecule-1
DiO: 3,3’-dioctadecyloxacarbocyanine perchlorate
NP-ICG: Nanoparticles loaded with indocyanine green
