# Supplementary material for: Neutrophil-inspired photothermo-responsive drug delivery system for targeted treatment of bacterial infection and endotoxins neutralization
Source: Biomater Res. 2023 Apr 15;27:30. doi: 10.1186/s40824-023-00372-z (PMC10105932; doi:10.1186/s40824-023-00372-z)
Supplement: Supplementary file 1 — Additional file 1: Figure S1.UV-Vis spectra of different samples. Figure S2. Photothermal heatingcurves of NM-NP-ICG/RIF. (a) Photothermal heating curves of NM-NP-ICG/RIF (534 μg mL-1) under different NIR irradiation (808 nm) power densities. (b) Photothermal heating curves of NM-NP-ICG/RIF with different concentrationsunder NIR irradiation (808 nm, 1.5 W cm-2). Figure S3. DSC thermogramsof PLGA and NP-ICG/RIF nanoparticles. Figure S4. TEMimages of (a) NP-ICG/RIF and (b) NM-NP-ICG/RIF nanoparticles after photothermalheating. Scale bar: 200 nm. Figure S5. Rifampicin release profiles fromNM-NP-ICG/RIF at different temperatures in dark. Figure S6. In vitro binding of NM-NP-ICG/RIF to inflammatory HUVEC cells. (a) CLSM images (left) and flow cytometry analysis (right) of the binding of NMVs (50 μg mL-1) to HUVEC cells with or without LPS stimulation. (b) CLSM images (left) and flowcytometry analysis (right) of the binding of nanoparticles with or without NMVscoating to LPS-stimulated HUVEC cells. Scale bar: 200 μm. Figure S7. CLSM images of P. aeruginosa treated with control, RIF (8 μg mL-1), NP-ICG (15 μg mL-1 of ICG), NP-ICG/RIF (8 μg mL-1 of RIF, 15 μg mL-1 of ICG) and NM-NP-ICG/RIF (8 μg mL-1 of RIF,15 μg mL-1 of ICG) without laser, and stained with LIVE/DEADBacLight bacterial viability kit. Scale bar: 20 µm. Figure S8. Binding efficiency of NM-NP to LPS under differentconcentrations. (n = 3). Figure S9. Biocompatibility assessments of NM-NP-ICG/RIF. (a) Viability of HUVECs, HACAT and HEK 293T cells after incubation with NM-NP-ICG/RIF nanoparticles at different concentrations for 24 h. (n= 3). (b) Hemolysis of mouse red blood cells upon treatment with NM-NP-ICG/RIF nanoparticles at different concentrations. The PBS-treated group and 0.1% triton-treated group were used as negative and positive controls, respectively. (n = 3). Table S1. Loading content of ICG and RIF in different nanoparticles. Table S2. Polydispersity index (PDI) of different nanoparticles. [file 40824_2023_372_MOESM1_ESM.docx]

**Supporting information**


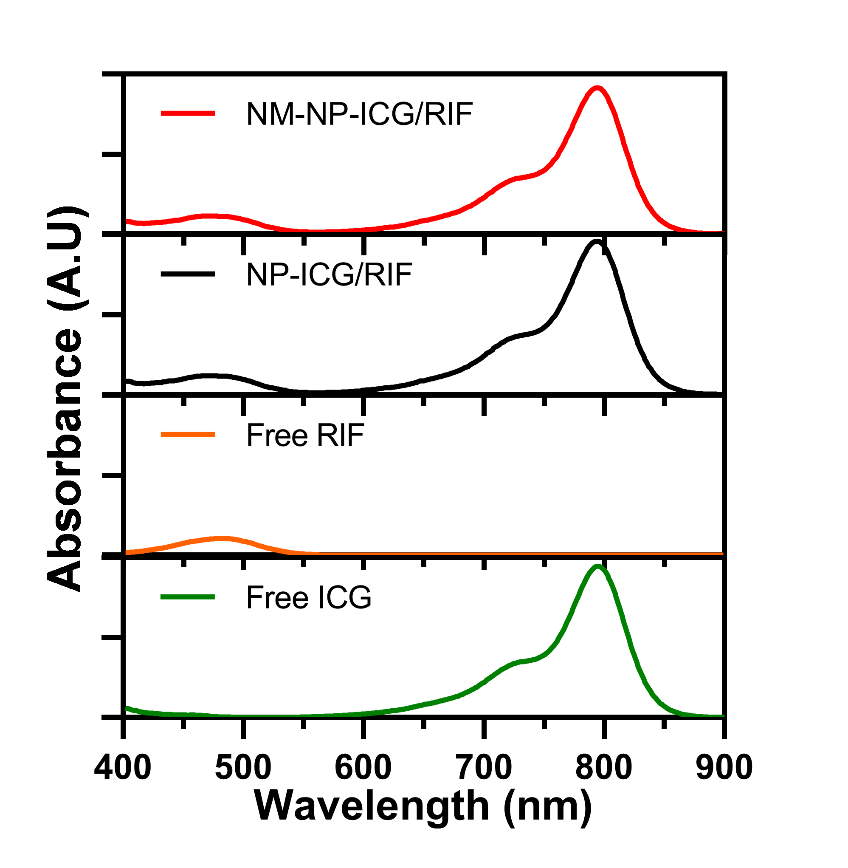


**Fig. S1.** UV-Vis spectra of different samples.


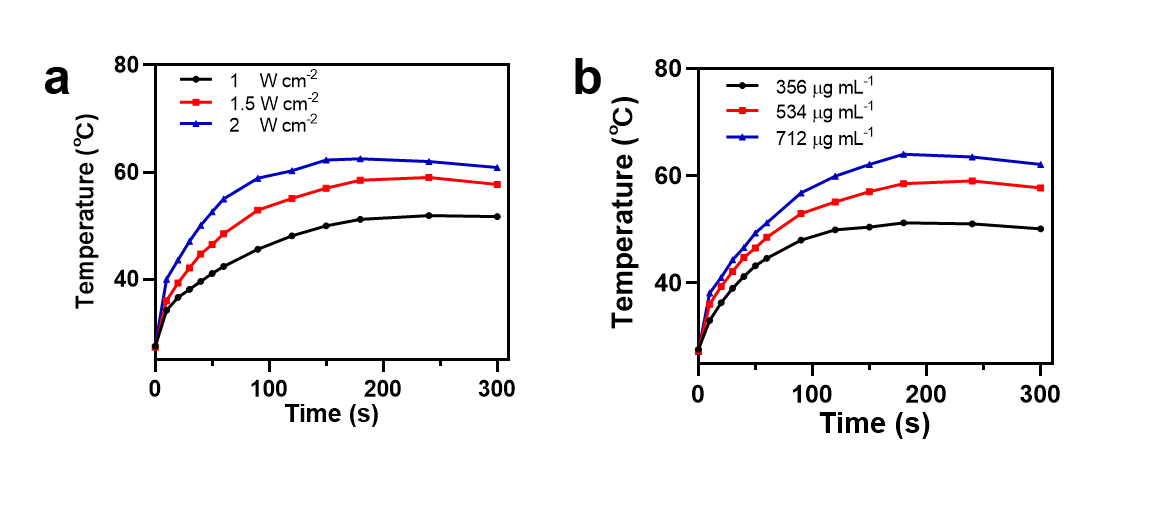


**Fig. S2.** Photothermal heating curves of NM-NP-ICG/RIF. (a) Photothermal heating curves of NM-NP-ICG/RIF (534 μg mL^-1^) under different NIR irradiation (808 nm) power densities. (b) Photothermal heating curves of NM-NP-ICG/RIF with different concentrations under NIR irradiation (808 nm, 1.5 W cm^-2^).


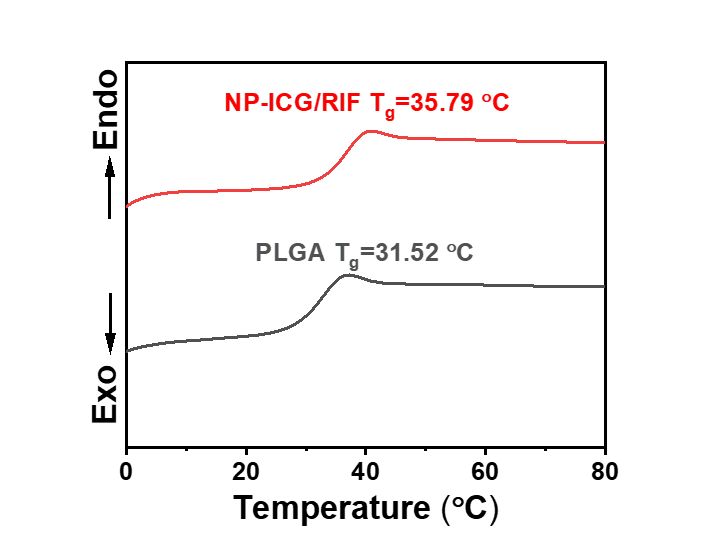


**Fig. S3**. DSC thermograms of PLGA and NP-ICG/RIF nanoparticles.


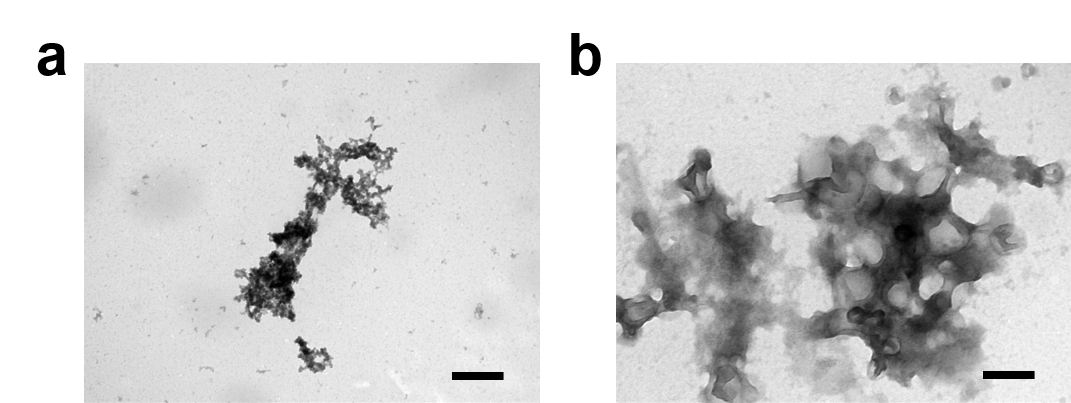


**Fig. S4.** TEM images of (a) NP-ICG/RIF and (b) NM-NP-ICG/RIF nanoparticles after photothermal heating. Scale bar: 200 nm.


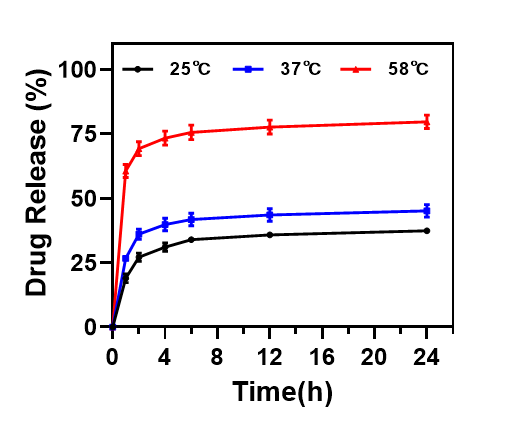


**Fig. S5**. Rifampicin release profiles from NM-NP-ICG/RIF at different temperatures in dark.


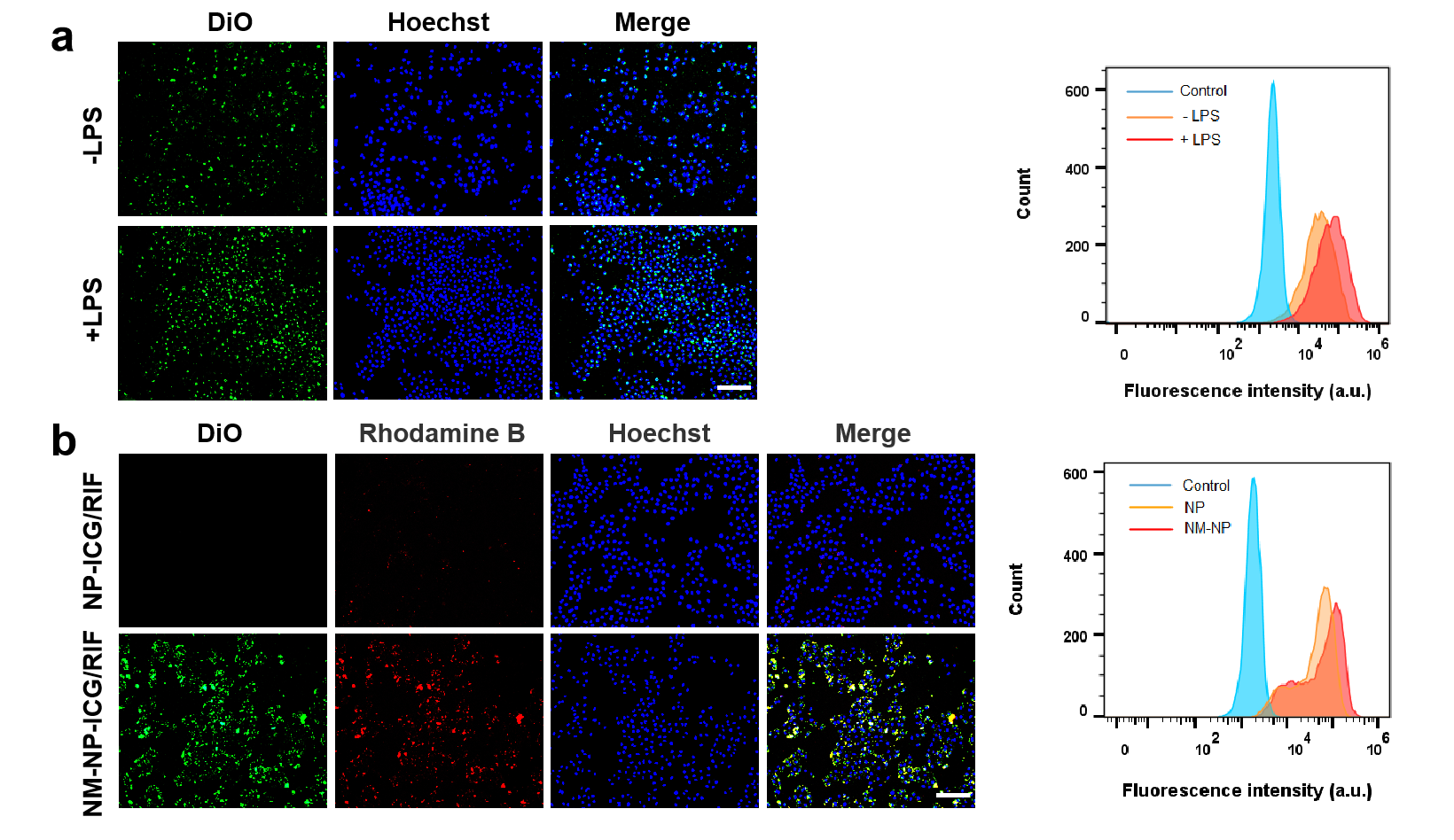


**Fig. S6.** *In vitro* binding of NM-NP-ICG/RIF to inflammatory HUVEC cells. (a) CLSM images (left) and flow cytometry analysis (right) of the binding of NMVs (50 μg mL^-1^) to HUVEC cells with or without LPS stimulation. (b) CLSM images (left) and flow cytometry analysis (right) of the binding of nanoparticles with or without NMVs coating to LPS-stimulated HUVEC cells. Scale bar: 200 μm.


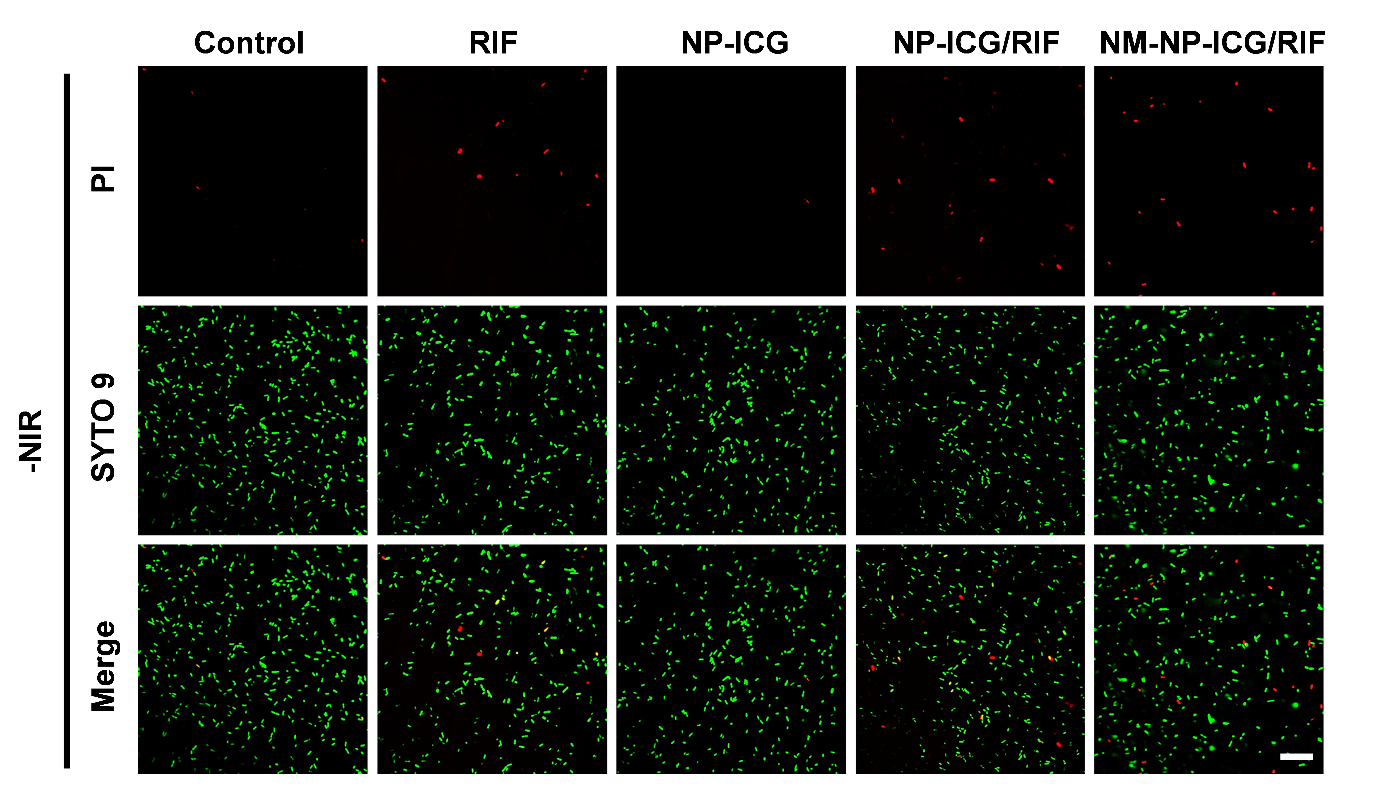


**Fig. S7.** CLSM images of *P. aeruginosa* treated with control, RIF (8 μg mL^-1^), NP-ICG (15 μg mL^-1^ of ICG), NP-ICG/RIF (8 μg mL^-1^ of RIF, 15 μg mL^-1^ of ICG) and NM-NP-ICG/RIF (8 μg mL^-1^ of RIF, 15 μg mL^-1^ of ICG) without laser, and stained with LIVE/DEAD BacLight bacterial viability kit. Scale bar: 20 µm.


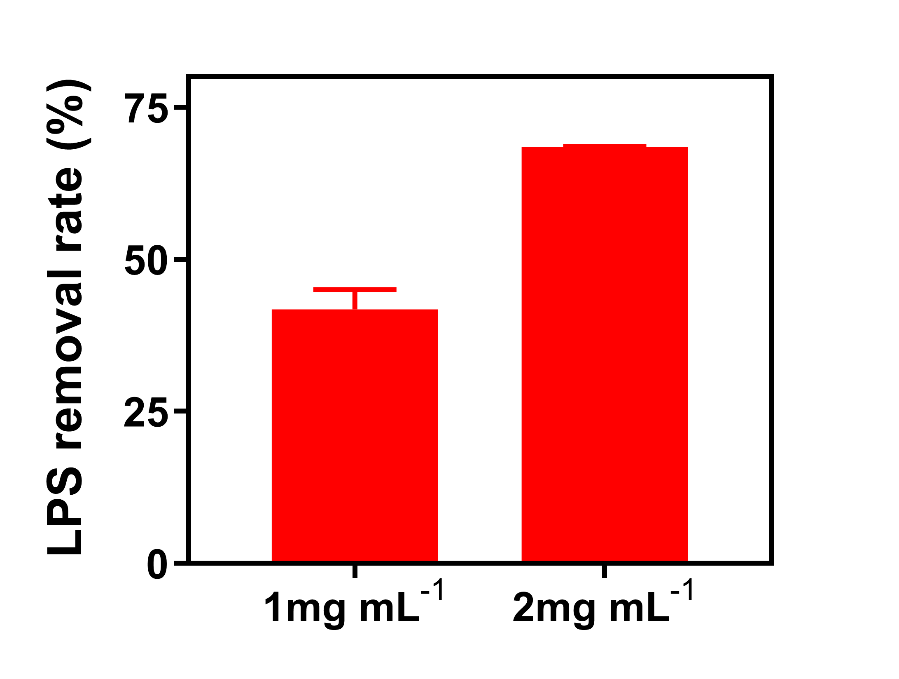


**Fig. S8.** Binding efficiency of NM-NP to LPS under different concentrations. (n = 3)


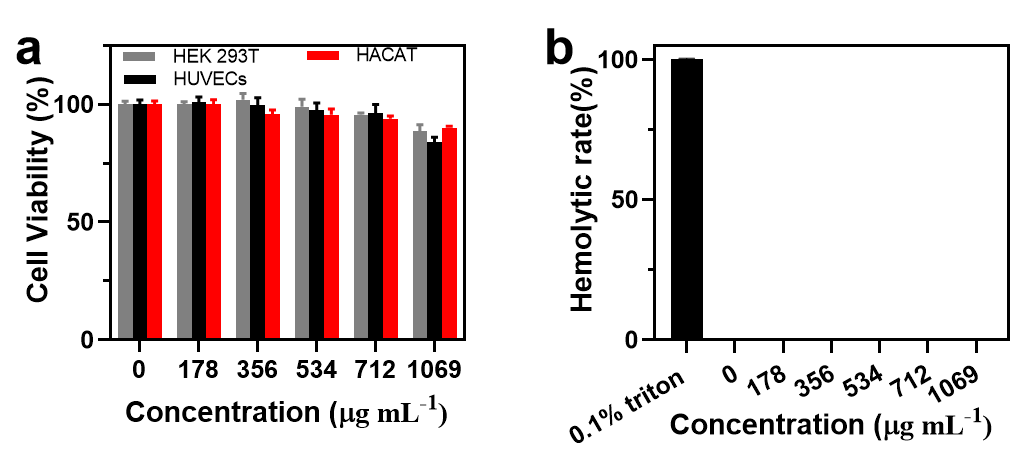


**Fig. S9.** Biocompatibility assessments of NM-NP-ICG/RIF. (a) Viability of HUVECs, HACAT and HEK 293T cells after incubation with NM-NP-ICG/RIF nanoparticles at different concentrations for 24 h. (n = 3) (b) Hemolysis of mouse red blood cells upon treatment with NM-NP-ICG/RIF nanoparticles at different concentrations. The PBS-treated group and 0.1% triton-treated group were used as negative and positive controls, respectively. (n = 3)

**Table S1.** Loading content of ICG and RIF in different nanoparticles

|  | NP-ICG/RIF | NM-NP-ICG/RIF |
| --- | --- | --- |
| ICG | 3.51% ± 0.23% | 2.81% ± 0.25% |
| RIF | 1.99% ± 0.15% | 1.68% ± 0.10% |

**Table S2.** Polydispersity index (PDI) of different nanoparticles

|  | NP-ICG/RIF | NMVs | NM-NP-ICG/RIF |
| --- | --- | --- | --- |
| PDI | 0.118 ± 0.025 | 0.201 ± 0.028 | 0.164 ± 0.036 |
